# Epstein Barr Virus (EBV) positive large B-cell lymphoma associated with breast implants: A case report^☆^

**DOI:** 10.1016/j.jpra.2025.08.036

**Published:** 2025-09-04

**Authors:** Liceth Lorena Patarroyo, Frank Álvarez Vásquez, Yasser Farid

**Affiliations:** a Plastic Surgeon - Bogotá, Colombia; b Doctor in Medicine– Bogotá, Cra 7b#134b-11 Bogotá DC, Colombia; c Department of Plastic and Reconstructive Surgery, Brugmann University Hospital, Brussels, Belgium

**Keywords:** Diffuse large B-cell lymphoma (DLBCL), Epstein-Barr Virus (EBV), Breast implant, Implant-associated lymphoma, Breast lymphoid neoplasia, Chronic inflammation, B-cell lymphoma, Primary breast lymphoma

## Abstract

Breast lymphoma associated with implants is an uncommon condition, with B-cell lymphomas representing a small subset. Among these, diffuse large B-cell lymphoma (DLBCL), particularly Epstein-Barr Virus (EBV)-positive, is extremely rare and poorly characterized. This lymphoma subtype differs from the more prevalent implant-associated anaplastic large-cell lymphoma. Chronic inflammation caused by prolonged contact between the implant and surrounding tissues is believed to create a microenvironment favorable for lymphoid proliferation and malignancy, though the underlying mechanisms remain speculative and require further exploration. We report the case of a female patient with Poland syndrome, a congenital anomaly involving chest wall deformities, who underwent left-sided breast reconstruction with an implant. She presented with implant rupture and capsular contracture, necessitating surgical removal of the implant and surrounding capsule. A total capsulectomy was performed, and reconstruction utilized the “no-touch” technique to reduce contamination. Intraoperative abnormalities of the periprosthetic capsule led to its submission for pathological analysis. Histopathological and immunohistochemical studies confirmed EBV-positive DLBCL. A PET/CT scan showed no hypermetabolic activity or extramammary involvement. Multidisciplinary evaluation concluded that conservative management with regular follow-up was appropriate, given the favorable prognosis. The patient remains under observation with no signs of recurrence. This case highlights the importance of considering lymphoma in patients with implants presenting with capsular changes or unexplained symptoms. Early diagnosis, involving thorough histopathological analysis and advanced imaging, is critical to guide management. Although EBV-positive implant-associated B-cell lymphomas generally have a good prognosis and rarely require aggressive treatment, their rarity underscores the need for continued documentation and research. Expanding our understanding of their pathogenesis and clinical behavior will help refine diagnostic and therapeutic approaches, improving patient outcomes.

**Section category:**

Reconstructive.

## Introduction

Primary breast lymphoma is a rare type of neoplasia that represents approximately 0.5 % of all malignant tumors at this site.^1^^,^^2^ This group of lymphomas is characterized by the presence of lymphoid neoplasms in breast tissue, with or without lymph node involvement, and without the presence of extramammary disease.^2^^,^^3^ Among lymphomas associated with breast implants, the majority are of T-cell type, with anaplastic large-cell lymphoma associated with breast implants (BIA-ALCL) being the most documented, with over 600 cases reported since its initial description in 1997. However, B-cell lymphomas, although much less common, have begun to gain attention in the medical literature.^1^, ^2^, ^3^

In this context, Epstein-Barr virus (EBV)-positive diffuse large B-cell lymphoma (DLBCL) associated with breast implants emerges as a significant clinical entity. This type of lymphoma may develop in an environment of chronic inflammation, fostered by the presence of breast implants. Bacterial biofilms or the constant friction between the implant and surrounding tissue may contribute to the abnormal proliferation of B cells, presenting a distinctive clinical profile that complicates its diagnosis and treatment.^4^

Existing literature indicates that while lymphomas associated with breast implants are predominantly T-cell in origin, isolated cases of B-cell lymphomas have been reported, with diffuse large B-cell lymphoma being the most representative among them. Of these, cases that are EBV-positive are particularly rare and highlight the need for further investigation into their etiology, prognosis, and clinical management.^5^^,^^6^

This article presents a case of EBV-positive DLBCL associated with a breast implant, contextualizing its clinical presentation and the existing literature on this poorly understood phenomenon. The collection and analysis of similar cases are essential for establishing more effective diagnostic and management protocols, as well as for enriching the understanding of the pathogenesis of these rare lymphoid entities.

## Case report

We present the case of a female patient in her sixth decade of life with a history of Poland syndrome, who had previously undergone left alloplastic breast reconstruction with an implant. Subsequently, she developed implant rupture and capsular contracture (Baker grade III), leading to a surgical intervention involving implant removal with total contained capsulectomy. During the procedure, the periprosthetic capsule exhibited atypical macroscopic characteristics, prompting its submission for histopathological examination. Simultaneously, unilateral breast reconstruction was performed using the “no-touch” technique with the placement of a new implant, with no intraoperative complications.

**Figure 1:**
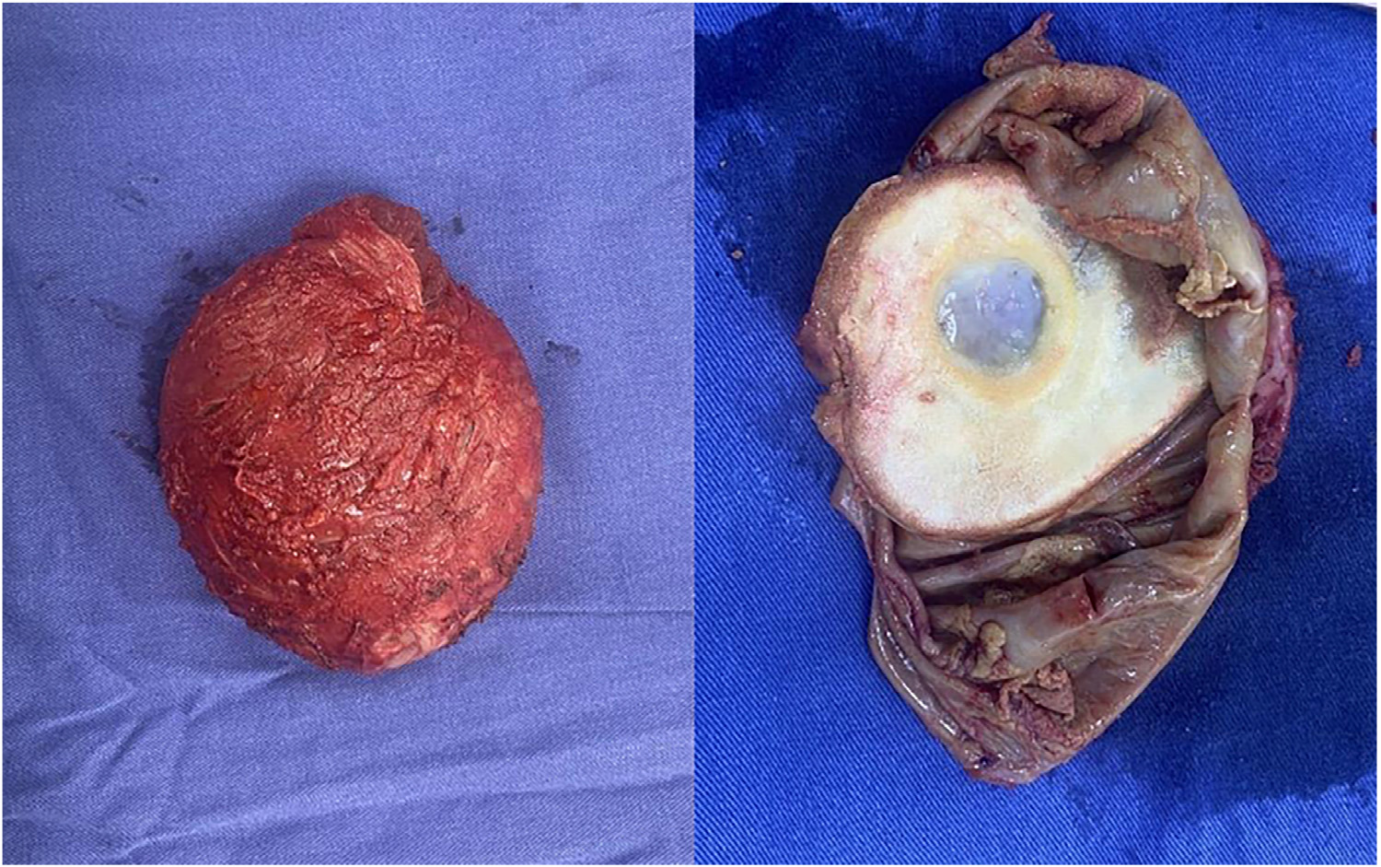
Macroscopic image of the left breast periprosthetic capsule.

In the outpatient setting, the pathology report described the periprosthetic capsule as having a smooth, shiny external surface with adherent adipose and muscular tissue. On sectioning, the capsule wall showed a thickness of up to 0.3 cm, with the internal surface displaying whitish calcified areas and a large yellowish-white friable plaque measuring 10 × 8 cm (Fig. 1).

Microscopic analysis revealed aggregates of atypical lymphoid cells with a neoplastic appearance, medium to large in size, confined to the capsule without infiltration into the represented breast tissue. These cells were surrounded by fibrinoid material and exhibited extensive necrosis, as well as calcifications within the prosthetic capsule. The pericapsular and breast tissue showed reactive lymphoplasmacytic infiltrates with foreign body giant cell reactions.

**Figure 2:**
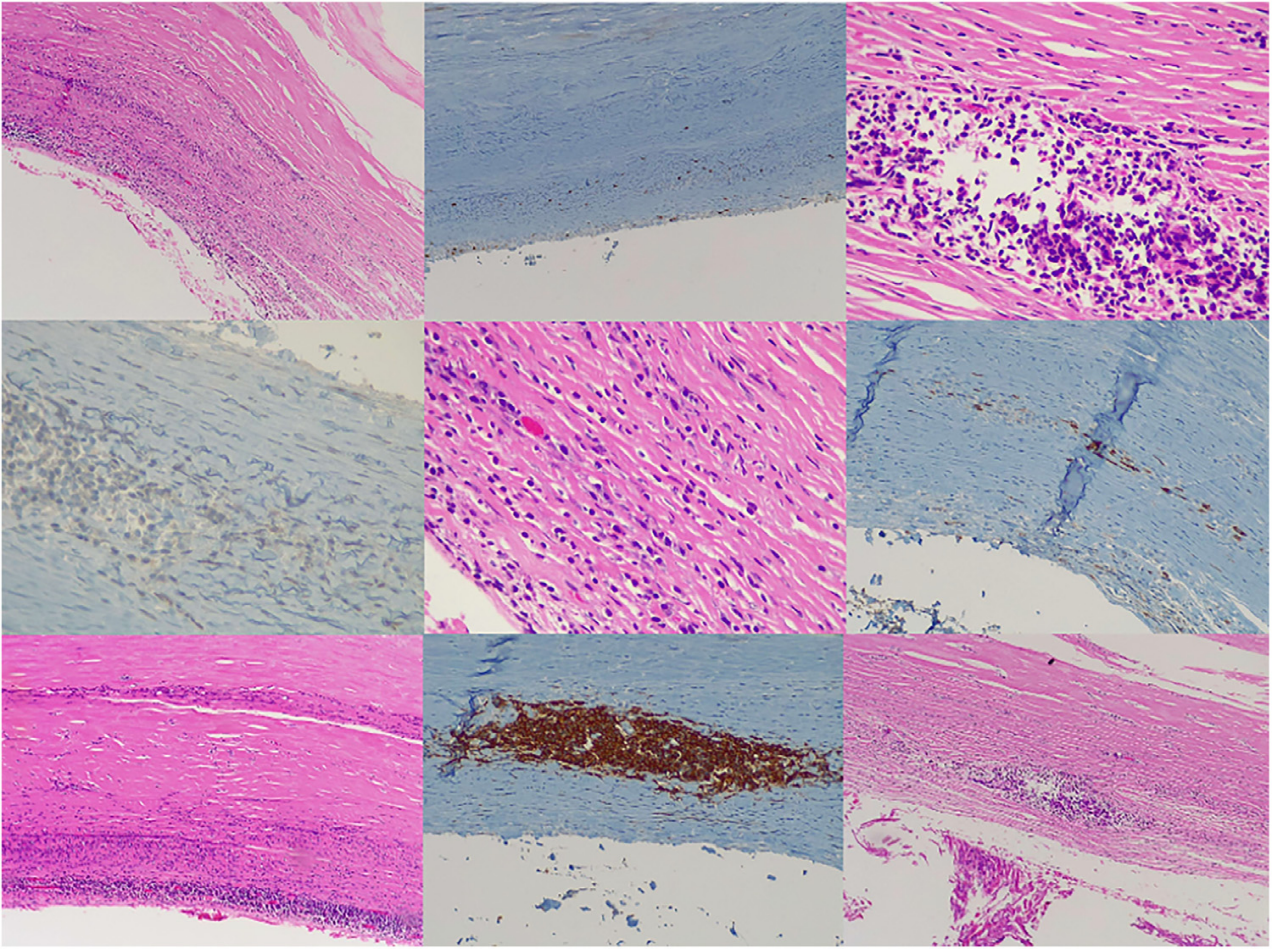
Microscopic image of the left breast periprosthetic capsule stained with hematoxylin-eosin, displaying representative histological features at different magnifications.

Immunohistochemistry confirmed the neoplastic lymphoid cells were positive for CD20, CD79a (weak), MUM1, CD30, LMP1, and BCL2, with a proliferation index (Ki-67) of up to 80 % in well-preserved areas. They were negative for CD10, CD5, CMYC, and BCL6. Plasma cells demonstrated polytypic staining for Kappa and Lambda light chains, while reactive T lymphocytes expressed CD3 and CD5 (Fig. 2).

The final diagnosis was: “Large B-cell lymphoma associated with Epstein-Barr virus (EBV) in the periprosthetic capsule.” The patient was referred to the hematology department, where a PET/CT scan ruled out adenopathy or infiltrative hypermetabolic clusters. A CT scan of the neck, chest, and abdomen confirmed the absence of disseminated disease.

Given the favorable prognosis of this lymphoma subtype, clinical follow-up without specific treatment was recommended. This type of lymphoma has been reported in the medical literature as an isolated finding, and most cases do not require additional therapy, as was observed in this case.

## Discussion

Primary breast lymphoma, although rare, represents a clinical entity that deserves attention, especially in the context of breast implants. This type of lymphoma comprises <0.5 % of all malignant breast neoplasms and is predominantly of B-cell origin.^2^^,^^6^^,^^7^ The association between lymphomas and breast prostheses has been widely documented, although most reported cases relate to anaplastic large-cell lymphomas of T-cell type (BIA-ALCL). However, B-cell lymphomas associated with implants, including those positive for Epstein-Barr virus (EBV), are even less common, with only a few cases reported in the literature.^8^, ^9^, ^10^ (see table) Table 1.

**Table 1:** Reported cases of EBV-positive B-cell lymphomas linked to breast implants.

Study	Number of cases	Patient age range	Time from implantation to diagnosis	Clinical presentation	Outcome
Morgan et al.^5^	2	57–62 years	5–12 years	Incidental finding post-mastectomyand painful palpable mass	Surgery + Chemotheraphy
Rodríguez-Pinilla et al.^6^	3	55–63 years	10–20 years	Fat necrosis, hematoma, localized symptoms	Surgical excision
Medeiros et al.^7^	8	39–68 years	4–22 years	Capsular contracture, late seroma, palpable mass and localized pain	Not specified
Vets et al.^8^	2	45–75 years	7–27 years	Rapidly growing mass, night sweats, weight loss, seroma and pain	Capsulectomy and implant removal; no chemotherapy; complete remission at 6 and 13 months
Brondeel et al.^9^	1	Middle-aged	7 years	Not specified	Surgical excision
Mescam et al.^10^	3	61–72 years	8–13 years	Incidental PET finding or capsular excision (no seroma/mass)	Watch-and-wait or limited chemo; all disease-free at 19–21 months follow-up
Malata et al.^11^	1	51 years	21 years	Recurrent severe capsular contracture	Observation (watch-and-wait); disease-free 2 years post-diagnosis
Martin de Bustamante et al.^12^	1	42 years	7 years	Bilateral capsular contracture (Grade III)	Surgical capsulectomy + prophylactic implant removal; disease-free

Summarized table of reported cases of EBV-positive B-cell lymphomas associated with breast implants based on available literature.

EBV-positive large B-cell lymphomas have been associated with environments of chronic inflammation, where EBV may play an oncogenic role. Previous studies have suggested that the persistence of inflammation in the tissues surrounding breast implants could favor the malignant transformation of B cells, establishing a microenvironment conducive to lymphoma development.^8^^,^^9^ This hypothesis is supported by literature linking EBV to other types of lymphomas in contexts of immunosuppression and chronic disease, suggesting that patients with breast implants may be at increased risk due to persistent inflammation and potential immune system alteration.^7^^,^^8^ The mechanisms involved in the pathogenesis of these lymphomas may include chronic stimulation of B cells by growth factors derived from inflammation, as well as the activation of signaling pathways associated with EBV-mediated oncogenesis, such as the NF-kB transcription factor pathway.^9^^,^^10^ This type of activation may be favored by the nature of the implant material and its capacity to induce a prolonged immune response.^6^^,^^7^^,^^8^

**Figure 3:**
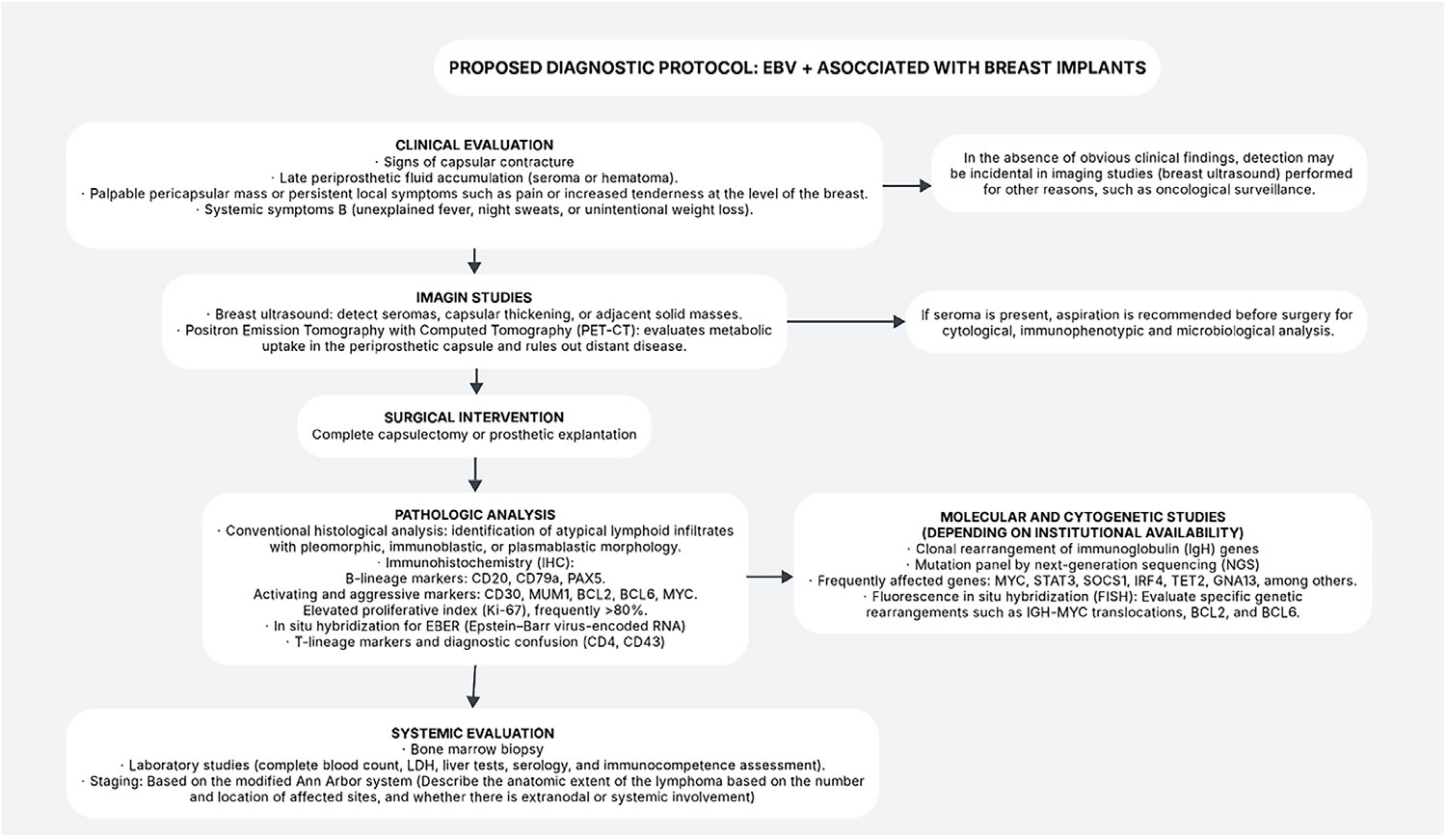
Flowchart of the diagnostic protocol for Epstein-Barr Virus (EBV)-positive breast implant-associated large B-cell lymphoma.

Additionally, correlations have been observed between B-cell lymphomas and macrophage activation syndrome (MAS), which has been described in cases of intravascular large B-cell lymphoma. The elevation of markers such as sIL-2R and ferritin in the serum of patients may serve as diagnostic and prognostic indicators, emphasizing the need for careful monitoring in patients with a history of breast implants.^7^^,^^8^^,^^10^ The scarcity of information and reported cases regarding B-cell lymphomas in the context of breast implants underscores the importance of increased vigilance and study (Fig. 3). Each new reported case will contribute to a broader understanding of the pathogenesis, management, and prognosis of this condition and will aid in establishing follow-up protocols for women with breast implants, particularly those with unusual clinical manifestations.^8^^,^^9^

Finally, the growing awareness of the relationship between B-cell lymphomas and breast implants, especially those associated with EBV, paves the way for future research that could explore not only the underlying mechanisms but also the possibility of preventive and therapeutic strategies in this patient population.^9^^,^^10^

## Conclusion

EBV-positive large B-cell lymphoma associated with breast implants is a rare but clinically significant condition that requires further investigation. While most implant-related lymphomas are T-cell in origin, emerging cases of EBV-positive B-cell lymphomas suggest a potential link between chronic inflammation, immune predisposition, and lymphomagenesis. Recognizing these cases is essential for refining diagnostic and treatment protocols. Clinicians should maintain a high index of suspicion in patients with unexplained symptoms, ensuring timely histopathological evaluation and imaging studies to facilitate early detection and improve patient outcomes.

## Funding

The authors received no financial support for the research, authorship, and publication of this article.

## Consent

Written informed consent was obtained from the patient for publication of this case report and accompanying images.

## Ethics approval

This study was performed in accordance with the principles of the Declaration of Helsinki. No Ethics Committee approval was needed.

## Declaration of competing interest

The authors declare that they have no known competing financial interests or personal relationships that could have appeared to influence the work reported in this paper.
